# Circular RNAs Are Promising Biomarkers in Liquid Biopsy for the Diagnosis of Non-small Cell Lung Cancer

**DOI:** 10.3389/fmolb.2021.625722

**Published:** 2021-05-31

**Authors:** Lanxiang Huang, Yuan Rong, Xuan Tang, Kezhen Yi, Jianyuan Wu, Fubing Wang

**Affiliations:** ^1^Department of Laboratory Medicine, Zhongnan Hospital of Wuhan University, Wuhan, China; ^2^Center for Single-Cell Omics and Tumor Liquid Biopsy, Zhongnan Hospital of Wuhan University, Wuhan, China; ^3^Clinical Trial Center, Zhongnan Hospital of Wuhan University, Wuhan, China; ^4^Wuhan Research Center for Infectious Diseases and Cancer, Chinese Academy of Medical Sciences, Wuhan, China

**Keywords:** circular RNAs, exosome, liquid biopsy, non-small cell lung cancer, biomarker

## Abstract

The high incidence and mortality of lung cancer make early detection of lung cancer particularly important. At present, the diagnosis of lung cancer mainly depends on diagnostic imaging and tissue biopsy. However, current diagnostics are not satisfactory owing to the low specificity and inability of multiple sampling. Accumulating evidence indicates that circular RNAs (circRNAs) play a critical role in cancer progression and are promising cancer biomarkers. In particular, circRNAs are considered novel specific diagnostic markers for non-small cell lung cancer (NSCLC). Liquid biopsy is an important method in the early diagnosis of cancer due to its high sensitivity and specificity, as well as the possibility of performing multiple sampling. circRNAs are stably present in exosomes and sometimes become part of circulating nucleic acids, making them ideal for liquid biopsy. In this review, we summarize the advances in the research on circRNAs in NSCLC, and also highlight their potential applications for NSCLC detection.

## Introduction

Cancer is a major public health problem worldwide. In China, lung cancer is one of the most common cancers and has the highest incidence and mortality rates among all cancers (Chen, 2015). The 5-year survival rate of patients with lung cancer is 54% when diagnosed at an early stage (Bach et al., 2012). However, this rate decreases to only 18% in patients with advanced cancer (Siegel et al., 2018). Non-small cell lung cancer (NSCLC), which is historically divided into adenocarcinoma, squamous cell carcinoma, and large cell carcinoma, accounts for approximately 80% of lung cancers. Using current diagnostics, NSCLC patients are usually diagnosed at the middle or late stages. To increase the survival rate of NSCLC patients, it is necessary to discover new biomarkers for its early diagnosis.

Precision oncology aims to improve the diagnosis and treatment of cancer (Ashley, 2016; Kumar-Sinha and Chinnaiyan, 2018). Studies on the comprehensive molecular signatures of cancers at the DNA, RNA, protein, and epigenetic levels deepen our understanding of the molecular events in different cancer types. Accurate identification of tumor subtypes and drug targets based on molecular signature profiling is a prerequisite for precision oncology (Heitzer et al., 2019). In the past few decades, molecular signatures have usually been identified by tissue biopsies. Histological examination is the gold standard for tumor diagnosis and grading (Chen, 2015). However, it is difficult to sample tumor tissues multiple times, and it is also difficult to conduct real-time tumor monitoring. In addition, cancer heterogeneity impedes the complete revelation of tumor information. Therefore, researchers have become increasingly interested in liquid biopsy, because liquid biopsy not only provides information equivalent to that provided by tissue biopsy, but also performs better than tissue biopsy in the minimally invasive, time-sensitive, and dynamic monitoring of cancers. With the advancement of research, liquid biopsy has played a significant role in the clinical testing of cancers.

In oncology, liquid biopsy refers to the sampling and measurement of analytes in various biological fluids (e.g., blood, urine, ascites, pleural effusions) (Siravegna et al., 2017). Peripheral blood analytes include circulating tumor cells (CTCs), circulating nucleic acids, circulating extracellular vesicles (EVs), tumor-educated platelets (TEPs), proteins, and metabolites. They can reveal several types of tumor information that are usually obtained by traditional tissue biopsy. Recent studies show that liquid biopsy could be a powerful tool for the early diagnosis of cancer (Cohen et al., 2018; Pantel and Alix-Panabićres, 2019).

Circular RNAs (circRNAs) are a type of endogenous non-coding RNAs (Shabaninejad et al., 2019; Borran et al., 2020; Naeli et al., 2020). Other types of non-coding RNAs include microRNAs (miRNAs) and long non-coding RNAs (lncRNAs) (Vafadar et al., 2019; Sadri Nahand et al., 2020; Razavi et al., 2021). circRNAs are stably present in exosomes or circulating RNAs, making them an important target for liquid biopsy. Significant circRNA expression has been found in patients with esophageal, gastric, liver, and lung cancer (Zhang et al., 2013; Jeck and Sharpless, 2014). In addition, circRNAs that carry tumor tissue information can be transported from tumor tissues to the blood by exosomes (Tang et al., 2018). Therefore, tumor tissue-derived exosomal circRNAs (exo-circRNAs)s could serve as novel circulating tumor biomarkers. This review summarizes the role of circRNAs in the progression of NSCLC, and discusses the feasibility of using circRNAs as NSCLC biomarkers.

## Characteristics and Functions of Circular RNAs

### Biogenesis and Characteristics of circRNAs

Circular RNAs were discovered by Nigro et al. (1991) in their analysis of human *DCC* gene sequencing. The 3′ and 5′ ends of circRNAs are not free, but covalently linked to each other. This linking occurs at a site flanked by canonical splice signals. To yield a circRNA, a splice donor must be linked to an upstream splice acceptor (Jeck et al., 2013; Memczak et al., 2013). This splicing product can be divided into two categories according to the differences in their structure and function: (1) circRNAs consisting exclusively of coding sequences and (2) circRNAs in which an intron is retained (exon-intron ciRNAs, EIciRNAs) (Xia et al., 2017).

circRNAs are widespread in eukaryotic cells. Evidence suggests that circRNAs have the following characteristics: (1) high stability. circRNAs are more stable than lncRNAs because of their circular structure. They are not easily degraded by exonucleases and thus have a longer half-life (Bachmayr-Heyda et al., 2015; Wu et al., 2019). (2) High abundance. Memczak et al. (2015) observed a higher expression of circRNAs relative to corresponding linear RNAs, as evidenced by the high circular-to-linear RNA ratio in the blood. In some cases, the ratio was more than 10-fold. (3) Evolutionary conservation. The sequences of circRNAs are evolutionarily conserved among different species (Jeck et al., 2013). (4) Specific expression. circRNAs are often expressed in a tissue- or developmental-stage-specific manner (Memczak et al., 2013).

### Functions of circRNAs

**FIGURE 1:**
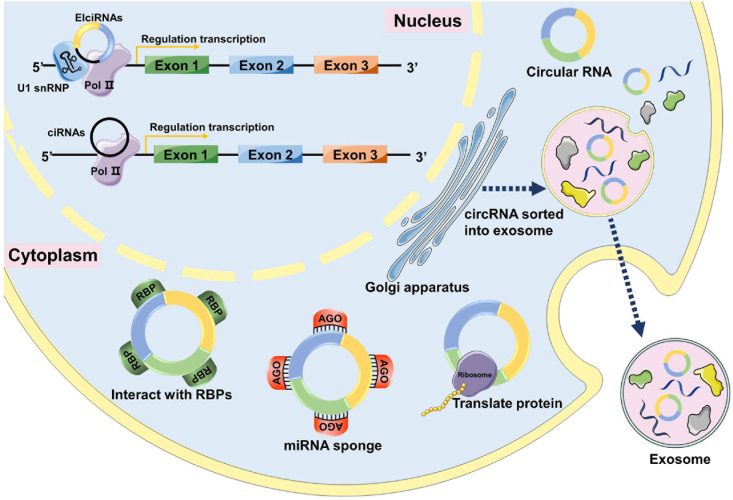
The functions of circRNAs. In the nucleus, circRNAs can interact with transcription complexes in the promoter region of their host gene to induce gene transcription by interacting with U1 snRNP. circRNAs can directly interact with transcription complexes on host genes to induce their transcription. In the cytoplasm, circRNAs can interact with RBPs and affect their functions and translocations. circRNAs can act as miRNA sponges to inhibit miRNA activity by interacting with miRNA-Ago2 complexes. Some circRNAs have protein-coding capacity and can encode proteins. circRNAs can be loaded into exosomes, released by donor cells, and enter recipient cells through endocytosis, thus modulating gene expression in recipient cells.

Previous studies have indicated that circRNAs have four major functions: act as miRNA sponges, interact with RNA-binding proteins, regulate gene transcription, and encode polypeptides (Figure 1).

#### Acting as miRNA Sponges

MicroRNA sponges are RNA transcripts containing multiple high-affinity binding sites that associate with and sequester specific miRNAs to prevent them from interacting with their target messenger RNAs (mRNAs). Some circRNAs contain a large number of miRNA binding sites and function as miRNA sponges to indirectly regulate gene expression. One of the representative circRNAs of this type is the ciRS-7 (Hansen et al., 2013). CirS7 has been shown to contain 470 conserved binding sites of miR7, thus it is considered a “sponge” of miR7 (Hansen et al., 2013). However, only a small fraction of circRNAs function as potential miRNA sponges in mammals (Chen, 2016).

#### Interacting With RNA-Binding Proteins

Several circRNAs can bind to RNA-binding proteins (RBPs) and function as RBP sponges. For example, circFoxo3 has been reported to interact with cyclin-dependent kinase 2 (Cdk2) and p21 to inhibit the G1-S transition in the cell cycle, resulting in cell cycle arrest (Du et al., 2016).

#### Regulating Gene Transcription

Circular RNAs can also regulate gene transcription. For instance, EIciRNAs (Li Z. et al., 2015) interact with U1 small nuclear RNA (snRNA) to enhance the expression of their parental genes in *cis* (Li Z. et al., 2015). circRNAs that regulate gene transcription are mainly located in the nucleus.

#### Encoding Polypeptides

Although circRNAs are non-coding RNAs, a few circRNAs encode polypeptides that exert regulatory functions. This feature of circRNAs increases the complexity of the transcriptome and proteome. circ-ZNF609 can be translated into proteins in a splicing-dependent and cap-independent manner (Legnini et al., 2017). Further analysis revealed that consensus m^6^A motifs were enriched in circRNAs, and a single m^6^A site was sufficient to drive translation initiation. The study also stated that the m^6^A-driven translation of circRNAs is widespread, and hundreds of endogenous circRNAs have translational potential (Yang et al., 2017).

## Identification and Validation of Circular RNAs

### Identification of circRNAs

RNA sequencing (RNA-seq) is an important method for genome-wide circRNA research. High-throughput sequencing allows for deeper sequencing and longer read lengths, making circRNA detection possible (Jeck and Sharpless, 2014). RNA-seq library preparation and bioinformatics analysis of RNA-seq data are the main challenges of high-throughput circRNA sequencing (Szabo and Salzman, 2016). RNA purification is crucial for library preparation and subsequent circRNA detection. Currently, circRNAs are typically purified by either poly(A)-selected or depleted ribosomal RNA (Szabo and Salzman, 2016; López-Jiménez et al., 2018). Various software packages, such as CIRI and circRNA_finder, can be used to analyze circRNAs from RNA-seq data, including 11 algorithms (Zeng et al., 2017). However, complicated data analysis methods and high cost severely limit the application of RNA-seq in circRNA detection. Compared with RNA-seq, a circRNA microarray is a more sensitive and efficient platform for circRNA identification. Combined with enzymatic linear RNA removal, a circRNA microarray uses unique circular junction-specific probes to enrich, capture, and quantify circRNAs with high sensitivity and specificity (Xu et al., 2011).

### Validation of circRNAs

Real-time fluorescent quantitative polymerase chain reaction (RT-qPCR) is essential for the verification of differentially expressed circRNAs after genome-wide sequencing or microarray analysis (Starke et al., 2015; Panda et al., 2017). In addition, RT-qPCR can conveniently quantify the expression of circRNAs used as biomarkers in clinical research (Guria et al., 2020). In a typical RT-qPCR analysis, total RNAs including linear RNAs (such as mRNAs and miRNAs) are extracted from cells, and linear RNAs are removed by ribosomal RNA (rRNA) consumption or ribonuclease R (RNase R) (an exoribonuclease) digestion. Next, complementary DNAs (cDNAs) of circRNAs are obtained by reverse transcription (RT). Finally, the fluorescent signal is accumulated and acquired by PCR amplification (Jeck and Sharpless, 2014). However, current methods for rRNA consumption and RNase R digestion cannot completely remove linear RNAs, especially linear RNAs with extensive secondary structures. Amplification of residual linear RNAs hinders quantitative and qualitative analysis of circRNAs (Panda et al., 2017). Furthermore, during the RT process, rolling circle amplification (RCA) using circRNAs as templates produces technical artifacts, leading to irreversible false-positive results (Szabo and Salzman, 2016).

Northern blotting enables the specific detection and characterization of circRNAs (Hansen et al., 2013; Zhang et al., 2014). Detection of specific circRNAs by northern blotting can be accomplished by short probes spanning the circular splice junction, or by longer probes covering as much as an entire circularized exon (Yang et al., 2018). Compared with RT-qPCR, northern blotting is laborious and time-consuming, but it is still an important tool for circRNA characterization (Schneider et al., 2018). circRNA *in situ* hybridization (RNA-ISH) can locate and quantify circRNAs by using specific probes that bind to back-spliced junction sites (Yang et al., 2020). However, it is also time-consuming and labor-intensive. In addition, RNA-ISH requires complex experimental steps and yields low detection signals. Therefore, advanced microscopic technology is required to enhance the signals to detectable levels.

Considering the growing interest in the comprehensive characterization of circRNAs, it is essential to develop better circRNA detection methods. The detection and imaging method based on catalyzed hairpin assembly (CHA) is a promising strategy. In CHA, two complementary nucleic acid hairpins are designed to kinetically hinder their spontaneous hybridization by embedding complementary regions in hairpin stems. In the presence of a target input chain, one hairpin is opened as a result of the toehold-mediated strand displacement, which further induces the assembly of the two hair clips. Finally, similar to a catalyst, the target chain is spontaneously replaced and recycled to trigger more hairpin assembly events (Jung and Ellington, 2014). The CHA-based method has high sensitivity and programmability. It has been proven to be a versatile tool for the detection of various molecules and events, including nucleic acids, small molecules, proteins, enzyme activity, metal ions, and cancer cells (Liu J. et al., 2019). Using this method, Jin et al. constructed a linear DNA nanostructure (LDN) to directly detect circRNAs in complex samples and even in cells (Jiao et al., 2020). They can accurately identify circRNAs without linear RNA-derived interference and image intracellular circRNAs *in situ*. Hence, CHA provides a simple, effective, and stable method for the detection and quantification of circRNAs. This may facilitate the clinical application of circRNAs as biomarkers.

## Circular RNAs in NSCLC

**FIGURE 2:**
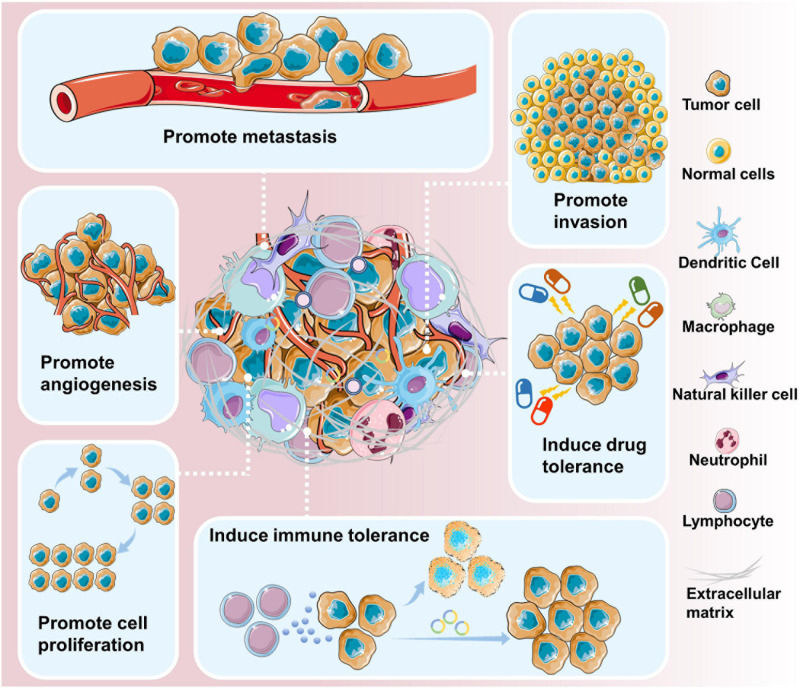
circRNAs are involved in cancer development and progression. circRNAs have been shown to contribute to various aspects of cancer progression, including promoting metastasis, promoting invasion, inducing drug tolerance, inducing immune tolerance, promoting cell proliferation, and promoting angiogenesis.

Circular RNAs are closely related to the initiation and development of cancer (Figure 2). Genome-wide analysis has revealed that circRNAs are differentially expressed in various cancer tissues and cell lines (Dou et al., 2016; Shang et al., 2016; Wan et al., 2016; Chen et al., 2017; Zhao et al., 2017). Cancer cell lines express a more diverse pattern of circRNAs than non-cancer cell lines (Hsiao et al., 2017; Liu X-X. et al., 2019).

Circular RNAs may be involved in the onset of lung cancer. By comparing the expression of circRNAs between tumor samples and paired adjacent normal tissues, researchers found that 357 circRNAs were dysregulated in patients with early-stage lung adenocarcinoma (Zhao et al., 2017). Indeed, high expression of certain circRNAs is positively correlated with the progression of lung cancer. For example, circ_0043278 promotes NSCLC cell proliferation, invasion, and migration by directly inhibiting miR-520f to increase the expression of ROCK1, CDKN1B, and AKT3 (Cui et al., 2019). Furthermore, there is a close correlation between the upregulated circRNA_100876, lymph node metastasis and tumor staging in NSCLC. The overall survival time of NSCLC patients with high circRNA_100876 expression was significantly shorter than that of patients with low circRNA_100876 expression (Yao et al., 2017).

Circular RNAs can also play a role in cancer inhibition (Wan et al., 2016). For instance, ectopic expression of *cir-ITCH* markedly elevated the expression of its parental cancer-suppressive gene *ITCH* and inhibited the proliferation of lung cancer cells. The molecular mechanism underlying this effect is that *cir-ITCH* acts as a sponge of oncogenic miR-7 and miR-214 to enhance *ITCH* expression, thus suppressing the activation of Wnt/β-catenin signaling.

In conclusion, some circRNAs are closely related to the pathogenesis of NSCLC. Therefore, they may serve as targets for NSCLC therapy.

## Circular RNAs as Promising Biomarkers for NSCLC

Circular RNAs can be easily and repeatedly detected in blood samples, making them promising biomarker candidates for human diseases (Memczak et al., 2015). Furthermore, the discovery of circRNAs in saliva indicates the presence of circRNAs in other body fluids (Bahn et al., 2015). Compared to traditional biomarkers found in tumor tissues, circRNAs in body fluids could be used as novel biomarkers in the more convenient and non-invasive “liquid biopsy” for the diagnosis of tumors at different stages. Until recently, efforts have been made to explore the significance of circRNAs as biomarkers for various types of cancers (Wang et al., 2016). At present, there are many studies on the application of circRNA expression in peripheral blood in the diagnosis of NSCLC, which proves the effectiveness of circRNA in the diagnosis of NSCLC (Table 1).

**TABLE 1:** circRNAs as biomarkers in liquid biopsy for the diagnosis of NSCLC.

**circRNA**	**Expression level**	**Intersection molecules and/or pathway**	**Effect**	**Sample**	**AUC**	**References**
circ_0008928	Up	miR-488/HK2 axis	Regulates cisplatin sensitivity, tumor progression, and glycolysis metabolism.	Serum exosomes		Shi et al., 2021
circ_PIP5K1A	Up	miR-101/ABCC1 axis	Regulates the progression of NSCLC and cisplatin sensitivity.	Serum and serum exosomes		Shao et al., 2021
circCDYL	Down	miR-185-5p/TNRC6A axis	Inhibited cell viability, proliferation, and induced apoptosis.	Plasma		Bian et al., 2021
hsa_circ_0014235	Up	miR-520a-5p/CDK4 axis	Promotes cisplatin chemoresistance and deteriorates the development of NSCLC.	Serum exosomes		Xu et al., 2020
has_circ_0060937	Up	–	Closely associated with bone metastasis in NSCLC.	Serum		Zhang J. et al., 2020
hsa_circ_0046264	Up	–	Notably associated with the patient’s age, tumor size, TNM stage, and lymph node metastasis.	Serum	0.915	Liu et al., 2020
circ-MEMO1	Up	miR-101-3p/KRAS axis	Promotes the progression and aerobic glycolysis of NSCLC.	Serum exosomes	0.760	Ding et al., 2020
circARHGAP10	Up	miR-638/FAM83F axis	Promotes proliferation, migration, invasion, and glycolysis.	Serum exosomes	–	Fang et al., 2020
circPVT1	Up	–	Associated with chemotherapy resistance.	Serum	–	Lu et al., 2020
hsa_circ_0002130	Up	miR-498/GLUT1/HK2/LDHA axis	Facilitates osimertinib resistance.	Serum exosomes	–	Ma et al., 2020
the panel of circ_0047921, circ_0056285, and circ_0007761	Up, Up, Down	–	Distinguishing early-stage NSCLC cases from healthy controls, chronic obstructive pulmonary disease controls, or tuberculosis controls.	Serum exosomes	0.919	Xian et al., 2020
circSATB2	Up	miR-326/FSCN1 axis	Promotes the proliferation, migration, and invasion of NSCLC cells.	Serum exosomes	–	Zhang N. et al., 2020
hsa_circRNA_012515	Up	–	May be a mechanism leading to gefitinib resistance in NSCLC patients.	Peripheral blood	–	Fu et al., 2020
hsa_circ_0134501 combined with hsa_circ_0109320	Down	–	Biomarker candidates for predicting the therapeutic effect of gefitinib in NSCLC.	Plasma	0.79	Liu Y.-T. et al., 2019
circ FECR1	Up	miR584-3p/ROCK1 axis	Predicts survival outcomes and predicts the response to chemotherapies.	Serum exosomes	–	Li et al., 2019
F-circEA	Exist	–	Could be a novel “liquid biopsy” biomarker to monitor the EML4-ALK fusion gene in NSCLC.	Plasma	–	Tan et al., 2018
hsa_circ_0102533	Up	–	Possesses an oncogenic property in the carcinogenesis.	Whole blood	0.744	Zhou et al., 2018
circFARSA	Up	miR-330-5p/FASN or miR-326/FASN axis	Promotes cell migration and invasion.	Plasma	0.71	Hang et al., 2018
hsa_circ_0013958	Up	miR-134/CCND1 axis	Promotes cell proliferation and invasion and inhibits cell apoptosis.	Plasma	0.794	Zhu et al., 2017

*TNM, tumor-node-metastasis; AUC, area under the ROC curve (AUC).*

**FIGURE 3:**
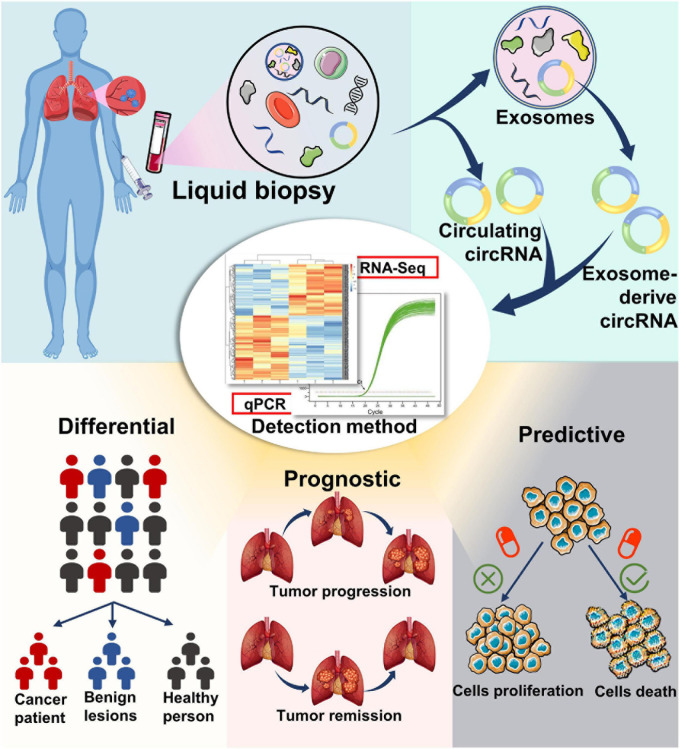
Potential application of circular RNAs as liquid biopsy biomarkers. circRNA biomarkers can be isolated from blood, as well as from the exosomes in blood. Detection and analysis of circRNAs can be completed using RNA-seq, qPCR, and other methods. circRNAs may serve as potential biomarkers for diagnosis, prognosis, and therapy selection of cancer.

In the past decades, numerous studies have investigated the expression profiles of miRNAs and lncRNAs in patients with NSCLC to assess if these RNA types can serve as biomarkers for the early detection of NSCLC (Wei and Zhou, 2016; Han and Li, 2018). However, the results remain controversial. Compared with other non-coding RNAs, circRNAs have superior features, such as higher stability, abundance, and evolutionary conservation, making them more suitable diagnostic biomarkers (Figure 3).

### Circular RNAs as Differential Diagnosis Biomarkers for NSCLC

At present, the most commonly used serum tumor markers for the diagnosis of NSCLC are embryonic antigen (CEA), squamous cell carcinoma antigen (SCCA), and cytokeratin 19 fragment (CYFRA21-1) (Ye et al., 2020). However, the sensitivity and specificity of detecting a single circulating biomarker in the diagnosis of lung cancer are not satisfactory, especially in the early stages (Kamel et al., 2019). circRNAs can stably exist in peripheral blood, and the detection of circRNAs expression in the blood has gradually become the focus of research in the diagnosis of NSCLC. Circulating miRNAs have shown potential advantages for early screening of NSCLC. A study found that circ_0047921, circ_0056285, and circ_0007761 in serum exosomes display significant diagnostic validity in distinguishing early-stage NSCLC cases from healthy controls, chronic obstructive pulmonary disease controls, and tuberculosis controls. In distinguishing NSCLC cases from healthy controls, the panel of the aforementioned circRNAs exhibited area under the curve (AUC) values of 0.926 [95% confidence interval (CI), 0.895–0.956] in the training set and 0.919 (95% CI, 0.877–0.962) in the validation set (Xian et al., 2020). A study on hsa_circ_0046264 showed that the area under the ROC curve (AUC) of hsa_circ_0046264 in serum was 0.915, the specificity was 0.957, and the sensitivity was 0.927 (Liu et al., 2020). The recent discovery of circRNAs in liquid biopsy samples suggests a novel and potentially useful tool for non-invasive NSCLC diagnosis.

### Circular RNAs as Prognostic Biomarkers for NSCLC

Prognostic biomarkers should provide information about the patient’s overall disease outcome, such as disease recurrence, metastasis, or progression, and may help identify high-risk cases that require active measures. circ_0067934 has been reported to function as an oncogenic circRNA in NSCLC by promoting the proliferation, migration, and invasion of NSCLC cells. High circ_0067934 expression is significantly associated with TNM stage, lymph node status, and distant metastasis, thus serving as a potential prognostic biomarker and therapeutic target for NSCLC (Wang and Li, 2018). Another study showed that by acting as a sponge for miR-1287 and regulating GAGE1 expression, circ_0016760 promoted the growth and metastasis of NSCLC cells, while inhibiting apoptosis. Moreover, the upregulation of circ_0016760 is associated with advanced TNM stages, lymph node metastasis, and poor prognosis in patients with NSCLC (Li Y. et al., 2018). Similarly, the upregulation of circRNA_100876 was tightly correlated with lymph node metastasis and advanced tumor stages in NSCLC. In addition, NSCLC patients with high circRNA_100876 expression had significantly shorter survival times than those with low circRNA_100876 expression. These observations strongly suggest the role of circRNAs as prognostic biomarkers.

### Circular RNAs as Predictive Biomarkers for Cancer Therapy

In addition, circRNAs may be useful for predicting tumor responsiveness to chemotherapy or different therapeutic approaches. Resistance to targeted therapy or immunotherapy is an ongoing problem in the successful treatment of NSCLC. It is increasingly necessary to develop biomarkers to monitor and predict the efficacy of targeted therapy or immunotherapy in patients with NSCLC. Recent studies suggested that circRNAs are likely to serve this purpose.

A study demonstrated that hsa_circ_0004015 increased the resistance of the NSCLC cell line HCC827 to gefitinib (an EGFR tyrosine kinase inhibitor) (Zhou et al., 2019). In addition, the EGFR-resistant LADC cell line H1975 expresses high levels of circRNA CCDC66 (Joseph et al., 2018). A high-throughput circRNA microarray showed that in comparison with parental cells, the paclitaxel-resistant lung adenocarcinoma cell line A549 had higher levels of 2,909 circRNAs and lower levels of 8,372 circRNAs (Xu et al., 2018). A recently identified circRNA, termed F-circEA, results from the fusion of the *ALK* gene with the *EML4* gene in NSCLC patients. For *EML4-ALK*-positive NSCLC patients, F-circEA could be both a novel diagnostic biomarker and a predictive biomarker of the response to targeted therapy (Tan et al., 2018). Another study showed that overexpression of circFGFR1 resulted in resistance of NSCLC to treatment with anti-programmed cell death protein-1 (PD-1) (Zhang et al., 2019). In general, these studies suggest that dysregulated circRNAs lead to resistance to targeted therapy and immunotherapy in NSCLC patients.

Circular RNAs also influence chemotherapy resistance in patients with NSCLC. It has been reported that circRNA cESRP1 enhances SCLC8 sensitivity to chemotherapy agents by inhibiting miR-93-5p (Huang et al., 2020). Downregulation of hsa_circ_0001946 increases NSCLC cell resistance to the chemotherapeutic drug cisplatin (Huang et al., 2019), whereas overexpression of circ-ABCB10 increases lung cancer resistance to cisplatin (Wu et al., 2020). Therefore, circRNAs may be used as biomarkers to predict the sensitivity or resistance to platinum-based chemotherapy.

### Exosomal circRNAs as NSCLC Biomarkers

Exosomes are tiny vesicles secreted by cells. They carry a variety of biomarkers such as DNA, RNA, proteins, and metabolites (Théry et al., 2002; Kahlert and Kalluri, 2013; Kumar et al., 2018). Exosomal contents are functional in recipient cells and highly variable depending on the origin of the cells. Cells can produce different exosomes under distinct physiological or pathological conditions (Fujita et al., 2016). Cancer cells produce more exosomes than their non-malignant counterparts (Rodríguez et al., 2014). Cancer cell-released exosomes induce phenotypic or functional alterations in recipient cells, thereby playing a role in cancer growth, angiogenesis, and metastasis (Kosaka et al., 2016).

Exosomes can mediate cell-to-cell communication *via* the direct exchange of genetic material between cells (Villarroya-Beltri et al., 2014). Exosomes can be isolated from diverse biofluids, such as blood (Caby et al., 2005), urine (Bryzgunova et al., 2016), and saliva (Ogawa et al., 2008). In recent years, circulating cancer-derived exosomes have emerged as promising biomarkers for monitoring cancer progression in non-invasive cancer diagnosis (Peinado et al., 2012; Xiao et al., 2012; Camacho et al., 2013; Zhou et al., 2014; Sundararajan et al., 2018). Studies on exosomal miRNAs and lncRNAs have contributed to the great progress in cancer screening, diagnosis, and prognostic evaluation (Liu et al., 2015; Li B. et al., 2018; Lee et al., 2019). However, our understanding of exo-circRNAs remains unclear.

Jae et al. first reported and validated circRNAs in human body fluids (Bahn et al., 2015). Later, another study confirmed abundant circRNAs in exosomes. RNA-seq analyses revealed the enrichment of circRNAs in the exosomes. It has been shown that more than 1,000 circRNAs are present in human serum exosomes (Li Y. et al., 2015), highlighting circRNAs as a novel class of stable RNA molecules in exosomes. Upon the uptake of EVs by neighboring or distant cells, exo-circRNAss can contribute to cell-to-cell communication. The existence of exo-circRNAs supports the idea that the expulsion of circRNAs from cells into the extracellular space, such as through EV release, is a probable mechanism by which circRNAs are cleared (Li Y. et al., 2015; Lasda and Parker, 2016).

Interestingly, serum exo-circRNAs might distinguish cancer patients from healthy individuals, illustrating the significant possibility of translating exo-circRNAs into cancer diagnostic biomarkers (Li Y. et al., 2015). A study found that circ-MEMO1 was higher in exosomes generated from the serum of NSCLC patients than in the healthy volunteers. The AUC reached a value of approximately 0.76, with a diagnostic sensitivity and specificity of 56.67 and 96%, respectively (95% CI 0.6259–0.8941) (Ding et al., 2020). Another study showed that circ_0008928 expression was increased in serum exosomes of cisplatin-resistant NSCLC patients compared with that in the cisplatin-sensitive NSCLC patients (Shi et al., 2021). In addition, a study showed that serum exosomal circFECR1 was associated with poor survival (*P* = 0.038) and clinical response to chemotherapy (Li et al., 2019). In summary, exo-circRNAs can be used as a promising biomarker for the diagnosis of NSCLC.

## Concluding Remarks and Perspectives

Liquid biopsy is a rapidly growing field, and advances in detection technology provide the possibility for the discovery and clinical translation of circulating cancer biomarkers. Although there has been extensive research on the discovery of circulating markers for cancer diagnosis, most biomarkers are still in the experimental stage. Before circulating biomarkers can be used in clinical practice, many biological, technical, and clinical challenges need to be addressed.

circRNAs have a clear functional role in the occurrence and development of NSCLC, and tumor-related differential expression has been detected in the peripheral blood of NSCLC patients. Due to the complexity of blood samples, it is necessary to develop a new methodology that can accurately extract and reproducibly measure NSCLC-related circulating circRNAs and conduct extensive verification in a large number of clinical samples to prove its clinical effectiveness. Furthermore, a study reported that it is obvious that the “multiple biomarker profile” has higher sensitivity and specificity than a single biomarker (Gulati et al., 2014). In the future, it is expected to improve the accuracy of circRNA in the diagnosis, prognosis, and prediction of NSCLC through the beneficial combination of circRNAs and different levels of other molecules (combining genome, transcriptome, and proteome). The transition from a single-biomarker view to a multi-biomarker view will help promote the development of liquid biopsy.

Circular RNAs have potential applications in both laboratory and clinical cancer research. However, there are still many questions regarding circRNAs. One major question is whether the products of endogenous circRNAs have specific functions in eukaryotic cells. Another interesting question is how circRNAs are degraded. To answer these questions, circRNAs should be studied in depth.

Moreover, circRNAs may be considered as novel therapeutic targets. Although there are no such reports to date, the effects of circRNAs on lung cancer proliferation, metastasis, and drug resistance indicate potential for targeting of circRNAs in anti-cancer therapy (Chi et al., 2019; Dong et al., 2019; Tian et al., 2019). For example, it is possible to deliver small interfering RNAs (siRNAs) or vectors into the body to target certain circRNAs in cancer patients. In addition, research on circRNA biogenesis might identify an approach to regulate the upstream pathways of circRNA expression (Verduci et al., 2019).

Taken together, the ultimate goal of circRNA research is to develop new effective diagnostic or therapeutic strategies for cancer treatment. With the advancement of elaborate research, our understanding of circRNAs will significantly contribute to cancer prevention, diagnosis, and treatment in the coming decades.

## Author Contributions

LH wrote the manuscript. YR conceived the review and performed bibliographic research. XT and KY prepared the figures and table. JW and FW contributed to the critical interpretation of the manuscript. All authors read and approved the final version of the manuscript.

## Conflict of Interest

The authors declare that the research was conducted in the absence of any commercial or financial relationships that could be construed as a potential conflict of interest.

## Abbreviations

circRNAs: circular RNAs
NSCLC: non-small cell lung cancer
CTCs: circulating tumor cells
EVs: extracellular vesicles
TEPs: tumor-educated platelets
miRNAs: microRNAs
lncRNAs: long non-coding RNAs
mRNAs: messenger RNAs
RBPs: RNA-binding proteins
Cdk2: cyclin-dependent kinase 2
snRNA: small nuclear RNA
RNA-seq: RNA sequencing
RT-Qpcr: real-time fluorescent quantitative polymerase chain reaction
rRNA: ribosomal RNA
RNase R: ribonuclease R
cDNAs: complementary DNAs
RT: reverse transcription
RCA: rolling circle amplification
RNA-ISH: RNA *in situ* hybridization
CHA: catalyzed hairpin assembly
LDN: linear DNA nanostructure
CEA: embryonic antigen
SCCA: squamous cell carcinoma antigen
CYFRA21-1: cytokeratin 19 fragment
ROC: receiver operating characteristic
AUC: area under the ROC curve
CI: confidence interval
PD-1: programmed cell death protein-1
exo-circRNAs: exosomal circRNAs
siRNA: small interfering RNA.

## References

[B1] Ashley E. A. (2016). Towards precision medicine. *Nat. Rev. Genet.* 17 507–522. 10.1038/nrg.2016.86 27528417

[B2] Bach P. B. Mirkin J. N. Oliver T. K. Azzoli C. G. Berry D. A. Brawley O. W. (2012). Benefits and harms of CT screening for lung cancer: a systematic review. *JAMA* 307 2418–2429. 10.1001/jama.2012.5521 22610500 PMC3709596

[B3] Bachmayr-Heyda A. Reiner A. T. Auer K. Sukhbaatar N. Aust S. Bachleitner-Hofmann T. (2015). Correlation of circular RNA abundance with proliferation–exemplified with colorectal and ovarian cancer, idiopathic lung fibrosis, and normal human tissues. *Sci. Rep.* 5:8057. 10.1038/srep08057 25624062 PMC4306919

[B4] Bahn J. H. Zhang Q. Li F. Chan T.-M. Lin X. Kim Y. (2015). The landscape of MicroRNA, Piwi-interacting RNA, and circular RNA in Human Saliva. *Clin. Chem.* 61 221–230. 10.1373/clinchem.2014.230433 25376581 PMC4332885

[B5] Bian W.-X. Xue F. Wang L.-Y. Xing X.-F. (2021). Circular RNA CircCDYL regulates proliferation and apoptosis in non-small cell lung cancer cells by sponging miR-185-5p and upregulating TNRC6A. *Cancer Manag. Res.* 13 633–642. 10.2147/CMAR.S280315 33531835 PMC7846864

[B6] Borran S. Ahmadi G. Rezaei S. Anari M. M. Modabberi M. Azarash Z. (2020). Circular RNAs: new players in thyroid cancer. *Pathol. Res. Pract.* 216 153217. 10.1016/j.prp.2020.153217 32987339

[B7] Bryzgunova O. E. Zaripov M. M. Skvortsova T. E. Lekchnov E. A. Grigor’eva A. E. Zaporozhchenko I. A. (2016). Comparative study of extracellular vesicles from the urine of healthy individuals and prostate cancer patients. *PLoS One* 11:e0157566. 10.1371/journal.pone.0157566 27305142 PMC4909321

[B8] Caby M. P. Lankar D. Vincendeau-Scherrer C. Raposo G. Bonnerot C. (2005). Exosomal-like vesicles are present in human blood plasma. *Int. Immunol.* 17 879–887. 10.1093/intimm/dxh267 15908444

[B9] Camacho L. Guerrero P. Marchetti D. (2013). MicroRNA and protein profiling of brain metastasis competent cell-derived exosomes. *PLoS One* 8:e73790. 10.1371/journal.pone.0073790 24066071 PMC3774795

[B10] Chen L.-L. (2016). The biogenesis and emerging roles of circular RNAs. *Nat. Rev. Mol. Cell Biol.* 17:205. 10.1038/nrm.2015.32 26908011

[B11] Chen S. Li T. Zhao Q. Xiao B. Guo J. (2017). Using circular RNA hsa_circ_0000190 as a new biomarker in the diagnosis of gastric cancer. *Clin. Chim. Acta* 466 167–171. 10.1016/j.cca.2017.01.025 28130019

[B12] Chen W. (2015). Cancer statistics: updated cancer burden in China. *Chin J Cancer Res.* 27 1. 25717219 10.3978/j.issn.1000-9604.2015.02.07 PMC4329178

[B13] Chi Y. Luo Q. Song Y. Yang F. Wang Y. Jin M. (2019). Circular RNA circPIP5K1A promotes non-small cell lung cancer proliferation and metastasis through miR-600/HIF-1α regulation. *J. Cell. Biochem.* 120 19019–19030. 10.1002/jcb.29225 31241217

[B14] Cohen J. D. Li L. Wang Y. Thoburn C. Afsari B. Danilova L. (2018). Detection and localization of surgically resectable cancers with a multi-analyte blood test. *Science* 359 926. 10.1126/science.aar3247 29348365 PMC6080308

[B15] Cui J. Li W. Liu G. Chen X. Gao X. Lu H. (2019). A novel circular RNA, hsa_circ_0043278, acts as a potential biomarker and promotes non-small cell lung cancer cell proliferation and migration by regulating miR-520f. *Artif. Cells Nanomed. Biotechnol.* 47 810–821. 10.1080/21691401.2019.1575847 30873868

[B16] Ding C. Xi G. Wang G. Cui D. Zhang B. Wang H. (2020). Exosomal Circ-MEMO1 promotes the progression and aerobic glycolysis of non-small cell lung cancer through targeting MiR-101-3p/KRAS Axis. *Front. Genet.* 11:962. 10.3389/fgene.2020.00962 33005174 PMC7483554

[B17] Dong Y. Xu T. Zhong S. Wang B. Zhang H. Wang X. (2019). Circ_0076305 regulates cisplatin resistance of non-small cell lung cancer via positively modulating STAT3 by sponging miR-296–5p. *Life Sci.* 239:116984. 10.1016/j.lfs.2019.116984 31647948

[B18] Dou Y. Cha D. J. Franklin J. L. Higginbotham J. N. Jeppesen D. K. Weaver A. M. (2016). Circular RNAs are down-regulated in KRAS mutant colon cancer cells and can be transferred to exosomes. *Sci. Rep.* 6:37982. 10.1038/srep37982 27892494 PMC5125100

[B19] Du W. W. Yang W. Liu E. Yang Z. Dhaliwal P. Yang B. B. (2016). Foxo3 circular RNA retards cell cycle progression via forming ternary complexes with p21 and CDK2. *Nucleic Acids Res.* 44 2846–2858. 10.1093/nar/gkw027 26861625 PMC4824104

[B20] Fang K. Chen X. Qiu F. Xu J. Xiong H. Zhang Z. (2020). Serum-derived exosomes-mediated circular RNA ARHGAP10 modulates the progression of non-small-cell lung cancer through the miR-638/FAM83F Axis. *Cancer Biother. Radiopharm.* 10.1089/cbr.2019.3534 [Epub ahead of print]. 32783691

[B21] Fu Y. Huang L. Tang H. Huang R. (2020). hsa_circRNA_012515 is highly expressed in NSCLC patients and affects its prognosis. *Cancer Manag. Res.* 12 1877–1886. 10.2147/CMAR.S245525 32210630 PMC7075336

[B22] Fujita Y. Yoshioka Y. Ochiya T. (2016). Extracellular vesicle transfer of cancer pathogenic components. *Cancer Sci.* 107 385–390. 10.1111/cas.12896 26797692 PMC4832849

[B23] Gulati S. Martinez P. Joshi T. Birkbak N. J. Santos C. R. Rowan A. J. (2014). Systematic evaluation of the prognostic impact and intratumour heterogeneity of clear cell renal cell carcinoma biomarkers. *Eur. Urol.* 66 936–948. 10.1016/j.eururo.2014.06.053 25047176 PMC4410302

[B24] Guria A. Sharma P. Natesan S. Pandi G. (2020). Circular RNAs-the road less traveled. *Front. Mol. Biosci.* 6:146. 10.3389/fmolb.2019.00146 31998746 PMC6965350

[B25] Han Y. Li H. (2018). miRNAs as biomarkers and for the early detection of non-small cell lung cancer (NSCLC). *J. Thorac. Dis.* 10 3119–3131. 10.21037/jtd.2018.05.32 29997981 PMC6006134

[B26] Hang D. Zhou J. Qin N. Zhou W. Ma H. Jin G. (2018). A novel plasma circular RNA circFARSA is a potential biomarker for non-small cell lung cancer. *Cancer Med.* 7 2783–2791. 10.1002/cam4.1514 29722168 PMC6010816

[B27] Hansen T. B. Jensen T. I. Clausen B. H. Bramsen J. B. Finsen B. Damgaard C. K. (2013). Natural RNA circles function as efficient microRNA sponges. *Nature* 495 384–388. 10.1038/nature11993 23446346

[B28] Heitzer E. Haque I. S. Roberts C. E. S. Speicher M. R. (2019). Current and future perspectives of liquid biopsies in genomics-driven oncology. *Nat. Rev. Genet.* 20 71–88. 10.1038/s41576-018-0071-5 30410101

[B29] Hsiao K.-Y. Lin Y.-C. Gupta S. K. Chang N. Yen L. Sun H. S. (2017). Noncoding effects of circular RNA CCDC66 promote colon cancer growth and metastasis. *Cancer Res.* 77 2339–2350. 10.1158/0008-5472.CAN-16-1883 28249903 PMC5910173

[B30] Huang M.-S. Liu J.-Y. Xia X.-B. Liu Y.-Z. Li X. Yin J.-Y. (2019). Hsa_circ_0001946 inhibits lung cancer progression and mediates cisplatin sensitivity in non-small cell lung cancer via the nucleotide excision repair signaling pathway. *Front. Oncol.* 9:508. 10.3389/fonc.2019.00508 31249811 PMC6582772

[B31] Huang W. Yang Y. Wu J. Niu Y. Yao Y. Zhang J. (2020). Circular RNA cESRP1 sensitises small cell lung cancer cells to chemotherapy by sponging miR-93-5p to inhibit TGF-β signalling. *Cell Death Differ.* 27 1709–1727. 10.1038/s41418-019-0455-x 31728016 PMC7206039

[B32] Jeck W. R. Sharpless N. E. (2014). Detecting and characterizing circular RNAs. *Nat. Biotechnol.* 32 453–461. 10.1038/nbt.2890 24811520 PMC4121655

[B33] Jeck W. R. Sorrentino J. A. Wang K. Slevin M. K. Burd C. E. Liu J. (2013). Circular RNAs are abundant, conserved, and associated with ALU repeats. *RNA* 19 141–157. 10.1261/rna.035667.112 23249747 PMC3543092

[B34] Jiao J. Xiang Y. Duan C. Liu Y. Li C. Li G. (2020). Lighting Up CircRNA using a linear DNA nanostructure. *Anal. Chem.* 92 12394–12399. 10.1021/acs.analchem.0c02146 32838512

[B35] Joseph N. A. Chiou S.-H. Lung Z. Yang C.-L. Lin T.-Y. Chang H.-W. (2018). The role of HGF-MET pathway and CCDC66 cirRNA expression in EGFR resistance and epithelial-to-mesenchymal transition of lung adenocarcinoma cells. *J. Hematol. Oncol.* 11:74. 10.1186/s13045-018-0557-9 29855336 PMC5984410

[B36] Jung C. Ellington A. D. (2014). Diagnostic applications of nucleic acid circuits. *Acc. Chem. Res.* 47 1825–1835. 10.1021/ar500059c 24828239 PMC4063332

[B37] Kahlert C. Kalluri R. (2013). Exosomes in tumor microenvironment influence cancer progression and metastasis. *J. Mol. Med. (Berl.)* 91 431–437. 10.1007/s00109-013-1020-6 23519402 PMC4073669

[B38] Kamel L. M. Atef D. M. Mackawy A. M. H. Shalaby S. M. Abdelraheim N. (2019). Circulating long non-coding RNA GAS5 and SOX2OT as potential biomarkers for diagnosis and prognosis of non-small cell lung cancer. *Biotechnol. Appl. Biochem.* 66 634–642. 10.1002/bab.1764 31077615

[B39] Kosaka N. Yoshioka Y. Fujita Y. Ochiya T. (2016). Versatile roles of extracellular vesicles in cancer. *J. Clin. Invest.* 126 1163–1172. 10.1172/JCI81130 26974161 PMC4811151

[B40] Kumar S. Michael I. J. Park J. Granick S. Cho Y. K. (2018). Cloaked exosomes: biocompatible, durable, and degradable encapsulation. *Small* 14:e1802052. 10.1002/smll.201802052 30024108

[B41] Kumar-Sinha C. Chinnaiyan A. M. (2018). Precision oncology in the age of integrative genomics. *Nat. Biotechnol.* 36 46–60. 10.1038/nbt.4017 29319699 PMC6364676

[B42] Lasda E. Parker R. (2016). Circular RNAs co-precipitate with extracellular vesicles: a possible mechanism for circRNA clearance. *PLoS One* 11:e0148407. 10.1371/journal.pone.0148407 26848835 PMC4743949

[B43] Lee Y. R. Kim G. Tak W. Y. Jang S. Y. Kweon Y. O. Park J. G. (2019). Circulating exosomal noncoding RNAs as prognostic biomarkers in human hepatocellular carcinoma. *Int. J. Cancer* 144 1444–1452. 10.1002/ijc.31931 30338850

[B44] Legnini I. Di Timoteo G. Rossi F. Morlando M. Briganti F. Sthandier O. (2017). Circ-ZNF609 Is a circular RNA that can be translated and functions in myogenesis. *Mol. Cell* 66 22–37.e9. 10.1016/j.molcel.2017.02.017 28344082 PMC5387670

[B45] Li B. Mao R. Liu C. Zhang W. Tang Y. Guo Z. (2018). LncRNA FAL1 promotes cell proliferation and migration by acting as a CeRNA of miR-1236 in hepatocellular carcinoma cells. *Life Sci.* 197 122–129. 10.1016/j.lfs.2018.02.006 29421439

[B46] Li L. Li W. Chen N. Zhao H. Xu G. Zhao Y. (2019). Exonic circular RNAs as a novel oncogenic driver to promote tumor metastasis in small cell lung cancer. *Clin. Cancer Res.* 25 1302–1317. 10.1158/1078-0432.CCR-18-1447 30429198

[B47] Li Y. Hu J. Li L. Cai S. Zhang H. Zhu X. (2018). Upregulated circular RNA circ_0016760 indicates unfavorable prognosis in NSCLC and promotes cell progression through miR-1287/GAGE1 axis. *Biochem. Biophys. Res. Commun.* 503 2089–2094. 10.1016/j.bbrc.2018.07.164 30103946

[B48] Li Y. Zheng Q. Bao C. Li S. Guo W. Zhao J. (2015). Circular RNA is enriched and stable in exosomes: a promising biomarker for cancer diagnosis. *Cell Res.* 25 981–984. 10.1038/cr.2015.82 26138677 PMC4528056

[B49] Li Z. Huang C. Bao C. Chen L. Lin M. Wang X. (2015). Exon-intron circular RNAs regulate transcription in the nucleus. *Nat. Struct. Amp. Mol. Biol.* 22:256. 10.1038/nsmb.2959 25664725

[B50] Liu J. Zhang Y. Xie H. Zhao L. Zheng L. Ye H. (2019). Applications of catalytic hairpin assembly reaction in biosensing. *Small* 15:1902989. 10.1002/smll.201902989 31523917

[B51] Liu W.-H. Ren L.-N. Wang X. Wang T. Zhang N. Gao Y. (2015). Combination of exosomes and circulating microRNAs may serve as a promising tumor marker complementary to alpha-fetoprotein for early-stage hepatocellular carcinoma diagnosis in rats. *J. Cancer Res. Clin. Oncol.* 141 1767–1778. 10.1007/s00432-015-1943-0 25724413 PMC11823797

[B52] Liu X.-X. Yang Y.-E. Liu X. Zhang M.-Y. Li R. Yin Y.-H. (2019). A two-circular RNA signature as a noninvasive diagnostic biomarker for lung adenocarcinoma. *J. Transl. Med.* 17:50. 10.1186/s12967-019-1800-z 30777071 PMC6380039

[B53] Liu Y.-T. Han X.-H. Xing P.-Y. Hu X.-S. Hao X.-Z. Wang Y. (2019). Circular RNA profiling identified as a biomarker for predicting the efficacy of Gefitinib therapy for non-small cell lung cancer. *J. Thorac. Dis.* 11 1779– 1787. 10.21037/jtd.2019.05.22 31285870 PMC6588778

[B54] Liu Z. H. Yang S. Z. Chen X. T. Shao M. R. Dong S. Y. Zhou S. Y. (2020). Correlations of hsa_circ_0046264 expression with onset, pathological stage and chemotherapy resistance of lung cancer. *Eur. Rev. Med. Pharmacol. Sci.* 24 9511–9521. 33015793 10.26355/eurrev_202009_23036

[B55] López-Jiménez E. Rojas A. M. Andrés-León E. (2018). “RNA sequencing and prediction tools for circular RNAs analysis,” in *Circular RNAs: Biogenesis and Functions*, ed. Xiao J. (Singapore: Springer Singapore), 17–33. 10.1007/978-981-13-1426-1_2 30259354

[B56] Lu H. Xie X. Chen Q. Cai S. Liu S. Bao C. (2020). Clinical significance of circPVT1 in patients with non-small cell lung cancer who received cisplatin combined with gemcitabine chemotherapy. *Tumori* 3008916209 41940. 10.1177/0300891620941940 32734834

[B57] Ma J. Qi G. Li L. A. (2020). Novel serum exosomes-based biomarker hsa_circ_0002130 facilitates osimertinib-resistance in non-small cell lung cancer by sponging miR-498. *Onco Targets Ther.* 13 5293–5307. 10.2147/OTT.S243214 32606748 PMC7293392

[B58] Memczak S. Jens M. Elefsinioti A. Torti F. Krueger J. Rybak A. (2013). Circular RNAs are a large class of animal RNAs with regulatory potency. *Nature* 495:333. 10.1038/nature11928 23446348

[B59] Memczak S. Papavasileiou P. Peters O. Rajewsky N. (2015). Identification and characterization of circular RNAs as a new class of putative biomarkers in human blood. *PLoS One* 10:e0141214. 10.1371/journal.pone.0141214 26485708 PMC4617279

[B60] Naeli P. Pourhanifeh M. H. Karimzadeh M. R. Shabaninejad Z. Movahedpour A. Tarrahimofrad H. (2020). Circular RNAs and gastrointestinal cancers: epigenetic regulators with a prognostic and therapeutic role. *Crit. Rev. Oncol. Hematol.* 145:102854. 10.1016/j.critrevonc.2019.102854 31877535 PMC6982584

[B61] Nigro J. M. Cho K. R. Fearon E. R. Kern S. E. Ruppert J. M. Oliner J. D. (1991). Scrambled exons. *Cell* 64 607–613. 10.1016/0092-8674(91)90244-S 1991322

[B62] Ogawa Y. Kanai-Azuma M. Akimoto Y. Kawakami H. Yanoshita R. (2008). Exosome-Like vesicles with dipeptidyl peptidase IV in human saliva. *Biol. Pharm. Bull.* 31 1059–1062. 10.1248/bpb.31.1059 18520029

[B63] Panda A. C. De S. Grammatikakis I. Munk R. Yang X. Piao Y. (2017). High-purity circular RNA isolation method (RPAD) reveals vast collection of intronic circRNAs. *Nucleic Acids Res* 45:e116. 10.1093/nar/gkx297 28444238 PMC5499592

[B64] Pantel K. Alix-Panabićres C. (2019). Liquid biopsy and minimal residual disease — latest advances and implications for cure. *Nat. Rev. Clin. Oncol.* 16 409–424. 10.1038/s41571-019-0187-3 30796368

[B65] Peinado H. Alećkovič M. Lavotshkin S. Matei I. Costa-Silva B. Moreno-Bueno G. (2012). Melanoma exosomes educate bone marrow progenitor cells toward a pro-metastatic phenotype through MET. *Nat. Med.* 18 883–891. 10.1038/nm.2753 22635005 PMC3645291

[B66] Razavi Z. S. Tajiknia V. Majidi S. Ghandali M. Mirzaei H. R. Rahimian N. (2021). Gynecologic cancers and non-coding RNAs: epigenetic regulators with emerging roles. *Crit. Rev. Oncol. Hematol.* 157:103192. 10.1016/j.critrevonc.2020.103192 33290823

[B67] Rodríguez M. Silva J. López-Alfonso A. López-Muřiz M. B. Peña C. Domínguez G. (2014). Different exosome cargo from plasma/bronchoalveolar lavage in non-small-cell lung cancer. *Genes Chromosomes Cancer* 53 713–724. 10.1002/gcc.22181 24764226

[B68] Sadri Nahand J. Moghoofei M. Salmaninejad A. Bahmanpour Z. Karimzadeh M. Nasiri M. (2020). Pathogenic role of exosomes and microRNAs in HPV-mediated inflammation and cervical cancer: a review. *Int. J. Cancer* 146 305–320. 10.1002/ijc.32688 31566705 PMC6999596

[B69] Schneider T. Schreiner S. Preußer C. Bindereif A. Rossbach O. (2018). “Northern blot analysis of circular RNAs,” in *Circular RNAs: Methods and Protocols*, eds Dieterich C. Papantonis A. (New York, NY: Springer New York), 119–133. 10.1007/978-1-4939-7562-4_10 29322445

[B70] Shabaninejad Z. Vafadar A. Movahedpour A. Ghasemi Y. Namdar A. Fathizadeh H. (2019). Circular RNAs in cancer: new insights into functions and implications in ovarian cancer. *J. Ovarian Res.* 12:84. 10.1186/s13048-019-0558-5 31481095 PMC6724287

[B71] Shang X. Li G. Liu H. Li T. Liu J. Zhao Q. (2016). Comprehensive circular RNA profiling reveals that hsa_circ_0005075, a new circular RNA biomarker, is involved in hepatocellular crcinoma development. *Medicine (Baltimore)* 95:e3811. 10.1097/MD.0000000000003811 27258521 PMC4900729

[B72] Shao N. Song L. Sun X. (2021). Exosomal circ_PIP5K1A regulates the progression of non-small cell lung cancer and cisplatin sensitivity by miR-101/ABCC1 axis. *Mol. Cell Biochem.* 476 2253–2267. 10.1007/s11010-021-04083-8 33570734

[B73] Shi Q. Ji T. Ma Z. Tan Q. Liang J. (2021). Serum exosomes-based biomarker circ_0008928 regulates cisplatin sensitivity, tumor progression, and glycolysis metabolism by miR-488/HK2 axis in cisplatin-resistant nonsmall cell lung carcinoma. *Cancer Biother. Radiopharm.* 10.1089/cbr.2020.4490 [Epub ahead of print]. 33661058

[B74] Siegel R. L. Miller K. D. Jemal A. (2018). Cancer statistics, 2018. *CA Cancer J. Clin.* 68 7–30. 10.3322/caac.21442 29313949

[B75] Siravegna G. Marsoni S. Siena S. Bardelli A. (2017). Integrating liquid biopsies into the management of cancer. *Nat. Rev. Clin. Oncol.* 14 531–548. 10.1038/nrclinonc.2017.14 28252003

[B76] Starke S. Jost I. Rossbach O. Schneider T. Schreiner S. Hung L.-H. (2015). Exon circularization requires canonical splice signals. *Cell Rep.* 10 103–111. 10.1016/j.celrep.2014.12.002 25543144

[B77] Sundararajan V. Sarkar F. H. Ramasamy T. S. (2018). The multifaceted role of exosomes in cancer progression: diagnostic and therapeutic implications [corrected]. *Cell. Oncol. (Dordr.)* 41 223–252. 10.1007/s13402-018-0378-4 29667069 PMC12995256

[B78] Szabo L. Salzman J. (2016). Detecting circular RNAs: bioinformatic and experimental challenges. *Nat. Rev. Genet.* 17 679–692. 10.1038/nrg.2016.114 27739534 PMC5565156

[B79] Tan S. Gou Q. Pu W. Guo C. Yang Y. Wu K. (2018). Circular RNA F-circEA produced from EML4-ALK fusion gene as a novel liquid biopsy biomarker for non-small cell lung cancer. *Cell Res.* 28 693–695. 10.1038/s41422-018-0033-7 29628502 PMC5993747

[B80] Tang W. Fu K. Sun H. Rong D. Wang H. Cao H. (2018). CircRNA microarray profiling identifies a novel circulating biomarker for detection of gastric cancer. *Mol. Cancer* 17:137. 10.1186/s12943-018-0888-8 30236115 PMC6147053

[B81] Théry C. Zitvogel L. Amigorena S. (2002). Exosomes: composition, biogenesis and function. *Nat. Rev. Immunol.* 2 569–579. 10.1038/nri855 12154376

[B82] Tian X. Zhang L. Jiao Y. Chen J. Shan Y. Yang W. (2019). CircABCB10 promotes nonsmall cell lung cancer cell proliferation and migration by regulating the miR-1252/FOXR2 axis. *J. Cell. Biochem.* 120 3765–3772. 10.1002/jcb.27657 30417418 PMC6587869

[B83] Vafadar A. Shabaninejad Z. Movahedpour A. Mohammadi S. Fathullahzadeh S. Mirzaei H. R. (2019). Long non-coding RNAs as epigenetic regulators in cancer. *Curr. Pharm. Des.* 25 3563–3577. 10.2174/1381612825666190830161528 31470781

[B84] Verduci L. Strano S. Yarden Y. Blandino G. (2019). The circRNA-microRNA code: emerging implications for cancer diagnosis and treatment. *Mol. Oncol.* 13 669–680. 10.1002/1878-0261.12468 30719845 PMC6441890

[B85] Villarroya-Beltri C. Baixauli F. Gutiérrez-Vázquez C. Sánchez-Madrid F. Mittelbrunn M. (2014). Sorting it out: regulation of exosome loading. *Semin. Cancer Biol.* 28 3–13. 10.1016/j.semcancer.2014.04.009 24769058 PMC4640178

[B86] Wan L. Zhang L. Fan K. Cheng Z.-X. Sun Q.-C. Wang J.-J. (2016). Circular RNA-ITCH suppresses lung cancer proliferation via inhibiting the Wnt/β-catenin pathway. *Biomed. Res. Int.* 2016:1579490. 10.1155/2016/1579490 27642589 PMC5013215

[B87] Wang F. Nazarali A. J. Ji S. (2016). Circular RNAs as potential biomarkers for cancer diagnosis and therapy. *Am. J. Cancer Res.* 6 1167–1176. 27429839 PMC4937728

[B88] Wang J. Li H. (2018). CircRNA circ_0067934 silencing inhibits the proliferation, migration and invasion of NSCLC cells and correlates with unfavorable prognosis in NSCLC. *Eur. Rev. Med. Pharmacol. Sci.* 22 3053–3060. 29863250 10.26355/eurrev_201805_15063

[B89] Wei M.-M. Zhou G.-B. (2016). Long non-coding rnas and their roles in non-small-cell lung cancer. *Genomics Proteomics Bioinformatics* 14 280–288. 10.1016/j.gpb.2016.03.007 27397102 PMC5093404

[B90] Wu J. Qi X. Liu L. Hu X. Liu J. Yang J. (2019). Emerging Epigenetic Regulation of Circular RNAs in Human Cancer. *Mol Ther Nucleic Acids.* 16 589–596. 10.1016/j.omtn.2019.04.011 31082792 PMC6517616

[B91] Wu Z. Gong Q. Yu Y. Zhu J. Li W. (2020). Knockdown of circ-ABCB10 promotes sensitivity of lung cancer cells to cisplatin via miR-556-3p/AK4 axis. *BMC Pulm. Med.* 20:10. 10.1186/s12890-019-1035-z 31931771 PMC6958770

[B92] Xia S. Y. Feng J. Lei L. J. Hu J. Xia L. J. Wang J. (2017). Comprehensive characterization of tissue-specific circular RNAs in the human and mouse genomes. *Brief Bioinform.* 18 984–992. 27543790 10.1093/bib/bbw081

[B93] Xian J. Su W. Liu L. Rao B. Lin M. Feng Y. (2020). Identification of three circular RNA cargoes in serum exosomes as diagnostic biomarkers of Non–small-cell lung cancer in the chinese population. *J. Mol. Diagnos.* 22 1096–1108. 10.1016/j.jmoldx.2020.05.011 32535085

[B94] Xiao D. Ohlendorf J. Chen Y. Taylor D. D. Rai S. N. Waigel S. (2012). Identifying mRNA, microRNA and protein profiles of melanoma exosomes. *PLoS One* 7:e46874. 10.1371/journal.pone.0046874 23056502 PMC3467276

[B95] Xu N. Chen S. Liu Y. Li W. Liu Z. Bian X. (2018). Profiles and bioinformatics analysis of differentially expressed circrnas in taxol-resistant non-small cell lung cancer cells. *Cell. Physiol. Biochem.* 48 2046– 2060. 10.1159/000492543 30099455

[B96] Xu W. Seok J. Mindrinos M. N. Schweitzer A. C. Jiang H. Wilhelmy J. (2011). Human transcriptome array for high-throughput clinical studies. *Proc. Natl. Acad. Sci. U.S.A.* 108 3707–3712. 10.1073/pnas.1019753108 21317363 PMC3048146

[B97] Xu X. Tao R. Sun L. Ji X. (2020). Exosome-transferred hsa_circ_0014235 promotes DDP chemoresistance and deteriorates the development of non-small cell lung cancer by mediating the miR-520a-5p/CDK4 pathway. *Cancer Cell Int.* 20:552. 10.1186/s12935-020-01642-9 33292236 PMC7672955

[B98] Yang H. Li X. Meng Q. Sun H. Wu S. Hu W. (2020). CircPTK2 (hsa_circ_0005273) as a novel therapeutic target for metastatic colorectal cancer. *Mol. Cancer* 19:13. 10.1186/s12943-020-1139-3 31973707 PMC6977296

[B99] Yang Y. Fan X. Mao M. Song X. Wu P. Zhang Y. (2017). Extensive translation of circular RNAs driven by N6-methyladenosine. *Cell Res.* 27:626. 10.1038/cr.2017.31 28281539 PMC5520850

[B100] Yang Y. Gao X. Zhang M. Yan S. Sun C. Xiao F. (2018). Novel Role of FBXW7 Circular RNA in repressing glioma tumorigenesis. *J. Natl. Cancer Inst.* 110 304–315. 10.1093/jnci/djx166 28903484 PMC6019044

[B101] Yao J.-T. Zhao S.-H. Liu Q.-P. Lv M.-Q. Zhou D.-X. Liao Z.-J. (2017). Over-expression of CircRNA_100876 in non-small cell lung cancer and its prognostic value. *Pathol. Res. Pract.* 213 453–456. 10.1016/j.prp.2017.02.011 28343871

[B102] Ye R. Tang R. Gan S. Li R. Cheng Y. Guo L. (2020). New insights into long non-coding RNAs in non-small cell lung cancer. *Biomed. Pharmacother.* 131:110775. 10.1016/j.biopha.2020.110775 33152934

[B103] Zeng X. Lin W. Guo M. Zou Q. (2017). A comprehensive overview and evaluation of circular RNA detection tools. *PLoS Comput. Biol.* 13:e1005420. 10.1371/journal.pcbi.1005420 28594838 PMC5466358

[B104] Zhang J. Mao W. Chen Z. Gu H. Lian C. (2020). Clinical significance of Has_circ_0060937 in bone metastasis of NSCLC. *Int. J. Gen. Med.* 13 1115–1121. 10.2147/IJGM.S279023 33209054 PMC7670089

[B105] Zhang N. Nan A. Chen L. Li X. Jia Y. Qiu M. (2020). Circular RNA circSATB2 promotes progression of non-small cell lung cancer cells. *Mol. Cancer* 19:101. 10.1186/s12943-020-01221-6 32493389 PMC7268724

[B106] Zhang P.-F. Pei X. Li K.-S. Jin L.-N. Wang F. Wu J. (2019). Circular RNA circFGFR1 promotes progression and anti-PD-1 resistance by sponging miR-381-3p in non-small cell lung cancer cells. *Mol. Cancer* 18:179. 10.1186/s12943-019-1111-2 31815619 PMC6900862

[B107] Zhang X.-O. Wang H.-B. Zhang Y. Lu X. Chen L.-L. Yang L. (2014). Complementary sequence-mediated exon circularization. *Cell* 159 134–147. 10.1016/j.cell.2014.09.001 25242744

[B108] Zhang Y. Zhang X. O. Chen T. Xiang J. F. Yin Q. F. Xing Y. H. (2013). Circular intronic long noncoding RNAs. *Mol. Cell* 51 792–806. 10.1016/j.molcel.2013.08.017 24035497

[B109] Zhao J. Li L. Wang Q. Han H. Zhan Q. Xu M. (2017). CircRNA expression profile in early-stage lung adenocarcinoma patients. *Cell. Physiol. Biochem.* 44 2138–2146. 10.1159/000485953 29241190

[B110] Zhou W. Fong M. Y. Min Y. Somlo G. Liu L. Palomares M. R. (2014). Cancer-secreted miR-105 destroys vascular endothelial barriers to promote metastasis. *Cancer Cell* 25 501–515. 10.1016/j.ccr.2014.03.007 24735924 PMC4016197

[B111] Zhou X. Liu H.-Y. Wang W.-Y. Zhao H. Wang T. (2018). Hsa_circ_0102533 serves as a blood-based biomarker for non-small-cell lung cancer diagnosis and regulates apoptosis in vitro. *Int. J. Clin. Exp. Pathol.* 11 4395–4404. 31949836 PMC6962954

[B112] Zhou Y. Zheng X. Xu B. Chen L. Wang Q. Deng H. (2019). Circular RNA hsa_circ_0004015 regulates the proliferation, invasion, and TKI drug resistance of non-small cell lung cancer by miR-1183/PDPK1 signaling pathway. *Biochem. Biophys. Res. Commun.* 508 527–535. 10.1016/j.bbrc.2018.11.157 30509491

[B113] Zhu X. Wang X. Wei S. Chen Y. Chen Y. Fan X. (2017). hsa_circ_0013958: a circular RNA and potential novel biomarker for lung adenocarcinoma. *FEBS J.* 284 2170–2182. 10.1111/febs.14132 28685964

